# Highly sensitive amperometric sensors based on laccase-mimetic nanozymes for the detection of dopamine†

**DOI:** 10.1039/d3ra07587g

**Published:** 2024-02-13

**Authors:** Olha Demkiv, Wojciech Nogala, Nataliya Stasyuk, Halyna Klepach, Taras Danysh, Mykhailo Gonchar

**Affiliations:** a Institute of Cell Biology, National Academy of Sciences of Ukraine Lviv 79005 Ukraine demkiv@yahoo.com; b Institute of Physical Chemistry, Polish Academy of Sciences 01-224 Warsaw Poland wnogala@ichf.edu.pl; c Drohobych Ivan Franko State Pedagogical University Drohobych 82100 Ukraine; d Institute of Blood Pathology and Transfusion Medicine, National Academy of Medical Sciences of Ukraine Lviv 79044 Ukraine

## Abstract

The current research presents novel sensors based on laccase-like mimetics for the detection of dopamine (DA). The synthesized laccase-like nanozymes (nAuCu, nPtCu, nCuMnCo, and nCoCuCe) were prepared by a simple hydrothermal method and exhibited an attractive catalytic activity toward DA. The developed amperometric sensors based on laccase nanozymes (nAuCu and nPtCu) are more stable, selective, and revealed a higher sensitivity (6.5-fold than the biosensor based on the natural fungal laccase from *Trametes zonata*). The amperometric sensors were obtained by modification of the glassy carbon electrodes (GCEs) with AuPt nanoparticles. Functionalization of the electrode surface by AuPt NPs resulted in increased catalytic activity of the laccase-like layer and higher sensitivity. Among studied configurations, the sensor containing nAuCu and nAuPt possesses a wide linear range for dopamine detection (10–170 μM), the lowest limit of detection (20 nM), and the highest sensitivity (10 650 ± 8.3 A M^−1^ m^−2^) at a low applied potential (+0.2 V *versus* Ag/AgCl). The proposed simple and cost-effective sensor electrode was used for the determination of DA in pharmaceuticals.

## Abstract 2

Novel nanozymes possesses the ability to mimic laccase. New amperometric biosensor for determination of dopamine was constructed.

## Graphical abstract

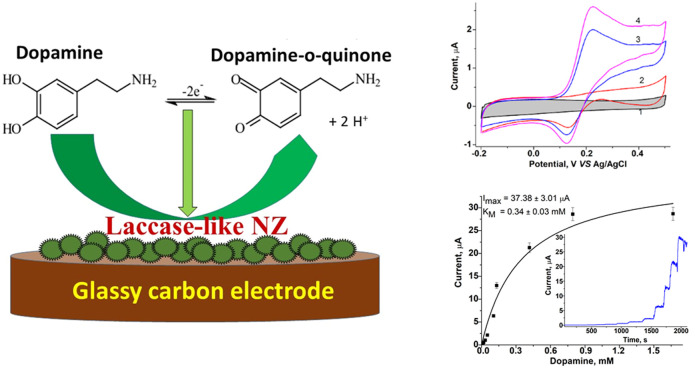

## Introduction

Dopamine (DA) is an important neurotransmitter in the central nervous, renal, hormonal and cardiovascular systems.^1^ In recent years, many factors have appeared that confirm its role as an important immunoregulatory factor.^2^ High levels of DA in blood indicate cardiotoxicity, leading to rapid heart rate, heart failure, and hypertension.^4^ Conversely, a low level of DA is associated with schizophrenia, depression, Alzheimer's and Parkinson's disease.^3,5^ Therefore, the level of DA in human serum and urine can be used as a diagnostic criterion for brain diseases and nervous system disorders.^2,3^ The fast detection of DA in human blood and urine is important not only for therapy, but also for early diagnostics of brain diseases.^6^

Different analytical methods such as enzymatic approaches, spectrometry, fluorescence, and chromatography are well known in the literature to detect DA.^5–7^ Although being very popular, the chromatography techniques suffer from several drawbacks, namely, the multi-stage sample preparation procedures are required and the maintenance costs are relatively high.^8^ To date, there is a great interest in the development of inexpensive electrochemical biosensors for the quantitative detection of DA, due to its ability to be easily oxidized on the surface of the electrode at specific potentials.^6,9^

It is known that amperometric biosensors based on natural enzymes, in particular, on laccases, are able to distinguish DA from its interferences, such as uric acid or ascorbic acid, as well as other neurotransmitters and molecules contained in real samples.^10^ However, due to the high cost of laccase and its non-sufficient stability (susceptibility to changes of pH and temperature during the storage) it is advantageous to substitute laccase by nanoparticles (NPs) possessing laccase-like activity.^11–15^

A number of low-cost laccase-like nanozymes NZs possessing high catalytic activity have been described and used for the development of DA-sensitive sensors, in particular, nanoparticles of Ni,^16^ Pd,^17^ Co,^18^ Au,^19^ Cu,^20^ different nanooxides and sulfides (FePt–Fe_3_O_4_, Fe_3_O_4_, Au@Fe_3_O_4_, ZnFe_2_O_4_, MoS_2_), some polymers, carbon nanomaterials (single-walled nanotubes, multi-walled nanotubes) and carbon-based materials functionalized with metal NPs.^21–26^ Detection of DA by electrochemical methods is not an easy task and is complicated by other redox biomolecules that can be oxidized at similar potentials, such as ascorbic acid or uric acid. Despite the numerous advantages of electrochemical sensors, their application to assay the neurotransmitter in human samples faces serious challenges due to presence of interfering compounds. For selective quantification of DA concentration, it is necessary to apply a material with a large specific surface area at which DA can be oxidized at low potential. In spite of this, there is an urgent need to synthesize new laccase mimicking materials with intrinsic redox activities and catalytic properties to overcome the limitations associated with the known electrochemical sensors.

The aim of the current research is to design DA selective sensors using metal hybrid NPs. The research focuses on the synthesis and characterization of nanocomposites and their application to sensors' development. The proposed nanoelectrodes are based on the of metal hybrid NPs were compared with laccase-based bioelectrodes.

The novelty of this paper lies in the synthesis of a wide range of hybrid metal nanoparticles, consisting of both transition and noble metals. It also covers their characterization and application in the analysis of DA at low working potentials. The application of laccase-mimicking NPs for electrode modification can increase the stability of the electrodes and reduce their cost, as well as improve the sensitivity of the sensors to the target analyte.

## Results and discussion

### Synthesis and characterization of the nanoparticles with laccase-like activity

The metallic nanoparticles were obtained by the hydrothermal approach (nAuCu, nPtCu, nCuMnCo) chemical reduction method (nCoCuCe), and the method of drop-synthesis by mixing aqueous solution of K_3_Fe(CN)_6_ and CoCl_2_ or CuSO_4_ (nCuHCF and nCoHCF). All the nanoparticles were evaluated for their Lac activity (LacNZs).^28^ The highest mimetic activities were found for four synthesized LacNZs, particularly, nCuMnCo, nCoCuCe, nAuCu and nPtCu (Fig. S1†), however these values were lower in some extent than for natural laccase from *Trametes zonata* in 3.0 (for nCuMnCo), 2.6 (for nCoCuCe), 1.26 (for nAuCu), 1.1 (for nPtCu) folds, respectively.

The morphology, size, and elemental composition of the synthesized LacNZs were analyzed using scanning electron microscopy (SEM) and energy dispersive X-ray spectroscopy (EDS). As shown in Fig. S2,† the nCuMnCo and nAuCu have three-dimensional spherical structures. nCuHCF and nCoCuCe had nanoflower, while nPtCu – spherical shape. The XRM images (Fig. S2†) demonstrated characteristic peaks corresponding to Cu, Pt, Au, and Fe, respectively.

As it is known, the size, shape and morphology of NZs plays a key role in regulating their catalytic activity.^29^ The activity of NZs are highly dependent on their composition, which can be regulated by chemical approaches such as doping or incorporating as well as on the particle size by influencing their surface active sites.^29,30^ Our studies demonstrate that the most active in solution laccase-like NZs, in particular, nAuCu, nPtCu and nCoCuCe (see Fig. S2†) are characterized nanoscale in sizes with a high specific surface area and probably, their high roughness of the surface is the one reason of high pseudo-enzymatic catalytic activity. The other reason of the catalytic activity of NZs is the composition of metal nanoclusters. The most electroactive nanoparticles contain Au, Cu, Pt, or a combination of these metals.^30^ The obtained LacNZs were further used for the development of NZs-based amperometric sensors for the detection of DA in pharmaceutical preparation.

### Electrochemical studies of the laccase mimetic-based amperometric sensors for the detection of DA

In the current work, different NZs-based sensors for DA detection were constructed. For the formation of chemoselective membrane, the NZs with composition: nAuCu, nPtCu, nCuMnCo, nCoCuCe, nCuHCF, and nCoHCF were used (Fig. S3†). Also, as a control, the biosensor based on natural fungal laccase *T. zonata* was constructed. The main principle of dopamine detection is based on the electrooxidation of DA to its corresponding *o*-quinone, followed by the detection of this product, at a low applied potential (0.2 V *versus* Ag/AgCl 3 M KCl).

**Fig. 1:**
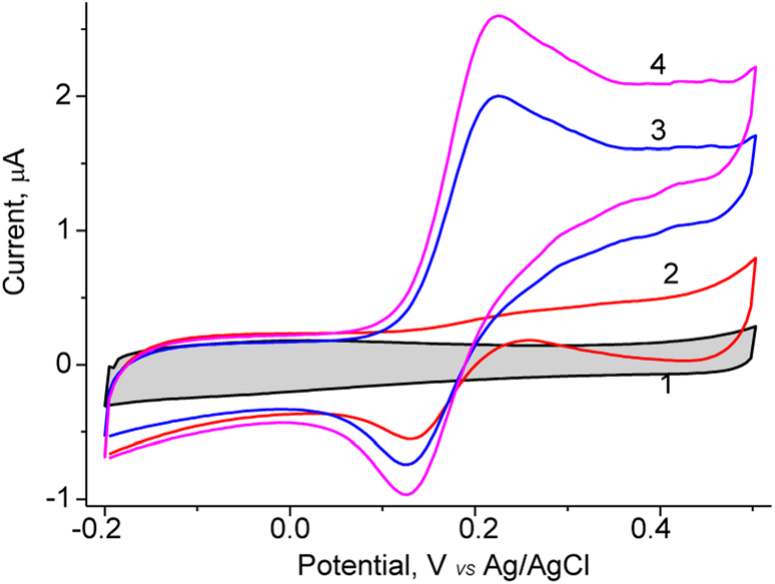
The cyclic voltammograms of the glassy carbon electrodes modified with nAuCu on addition of DA: (1)—0 mM; (2)—0.05 mM; (3)—0.1 mM; (4)—0.2 mM. Conditions: scan rate 25 mV s^−1^; Ag/AgCl (reference electrode) in 50 mM phosphate buffer, pH 7.0.

The obtained NPs were screened on their redox activity in the presence of DA using cyclic voltammetry. As shown Fig. 1, for the constructed sensor with architecture nAuCu/GCE the values of the anodic peak is caused by oxidation DA at the added concentrations from 0.05 to 0.2 mM at the working potential +200 mV (*vs.* Ag/AgCl).

The fabricated sensor with architecture nAuCu/GCE, the effect of pH on the DA oxidation in 0.05 M phosphate buffer in the pH range from 4.5 to 8.0 (Fig. S4†) was studied. The optimal catalytic activity of nAuCu was observed between pH 6.5 and 7.5. Thus, pH 7.0 was chosen as optimal for DA detection in further experiments. When comparing the catalytic activity of nAuCu nanozyme with natural laccase over a wide pH range, it was observed that natural laccase exhibits a higher activity between pH 3.5 and 6 with a maximum at pH 4.5 (data not shown).

**Fig. 2:**
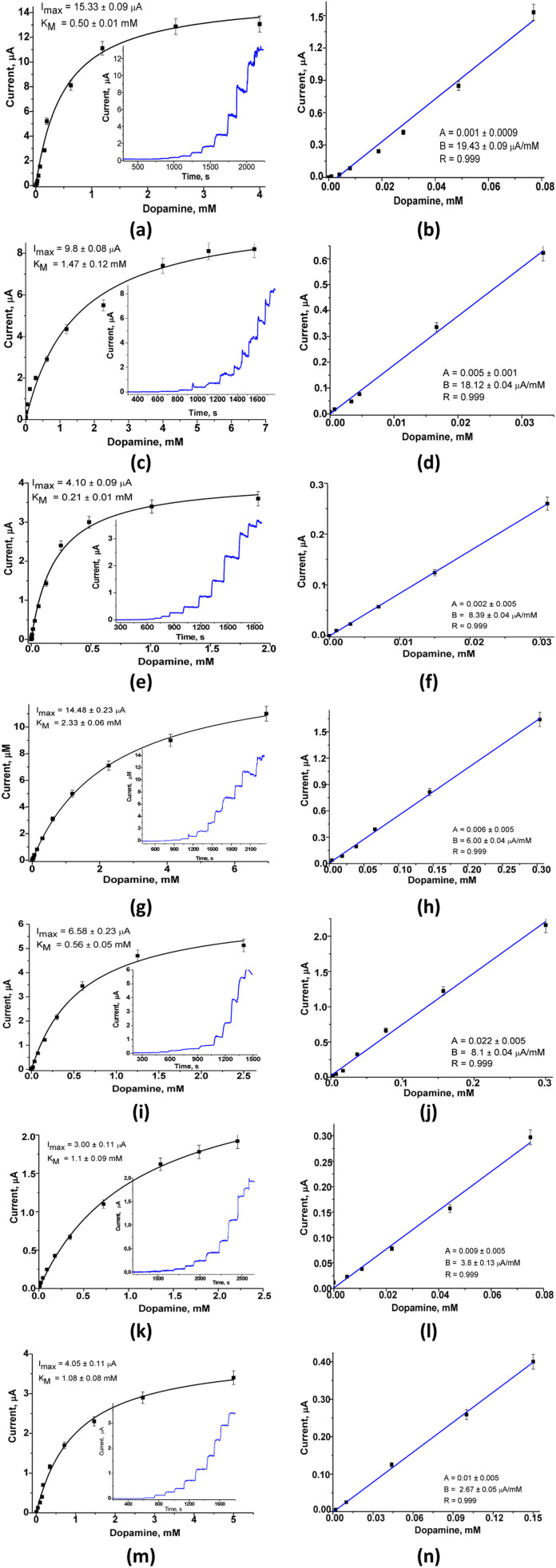
The chronoamperograms (a, c, e, i, k and m), and calibration curves (b, d, f, h, j, l and n) of the fabricated LacNZs-based sensors: nAuCu/GCE (a and b), nPtCu/GCE (c and d), nCuMnCo/GCE (e and f), nCoCuCe/GCE (g and h), nCuHCF/GCE (i and j), nCoHCF/GCE (k and l), and laccase/GCE biosensor (m and n) in PB (pH 7.0) at added different concentrations of DA. Working potential – +0.20 mV *versus* Ag/AgCl, 50 mM PB (pH 7.0).

For the constructed LacNZs-based sensors, namely: nAuCu/GCE, nPtCu/GCE, nCuMnCo/GCE, nCoCuCe/GCE, nCuHCF/GCE, and nCoHCF/GCE the main bioanalytical properties at working potential 0.2 V were investigated (Fig. 2).

The six sensors based on laccase mimetics (nAuCu, nPtCu, nCuMnCo, nCoCuCe, nCuHCF, and nCoHCF) reveal a higher sensitivity (approx. 2-fold) compared to the laccase-based biosensor (Table S1†). Among the tested sensors, only one (nAuCu/GCE) possess the highest sensitivity (2800 ± 1.89 A M^−1^ m^−2^, respectively), and a good linear range (0.4–80 μM) with the limit of detection 6 nM. Other sensors (nCoCuCe/GCE, nCuHCF/GCE, and nCoHCF/GCE) showed a lower sensitivity, but have a wider linear range – up to 300 μM (Fig. 2). The sensors with the highest sensitivity, namely, nAuCu/GCE and nPtCu/GCE were selected for further research on analysis of DA in real pharmaceutical samples.

### Modification of glassy carbon electrode by Au–Pt hybrid nanoparticles for fabrication of LacNZs-based sensors

**Fig. 3:**
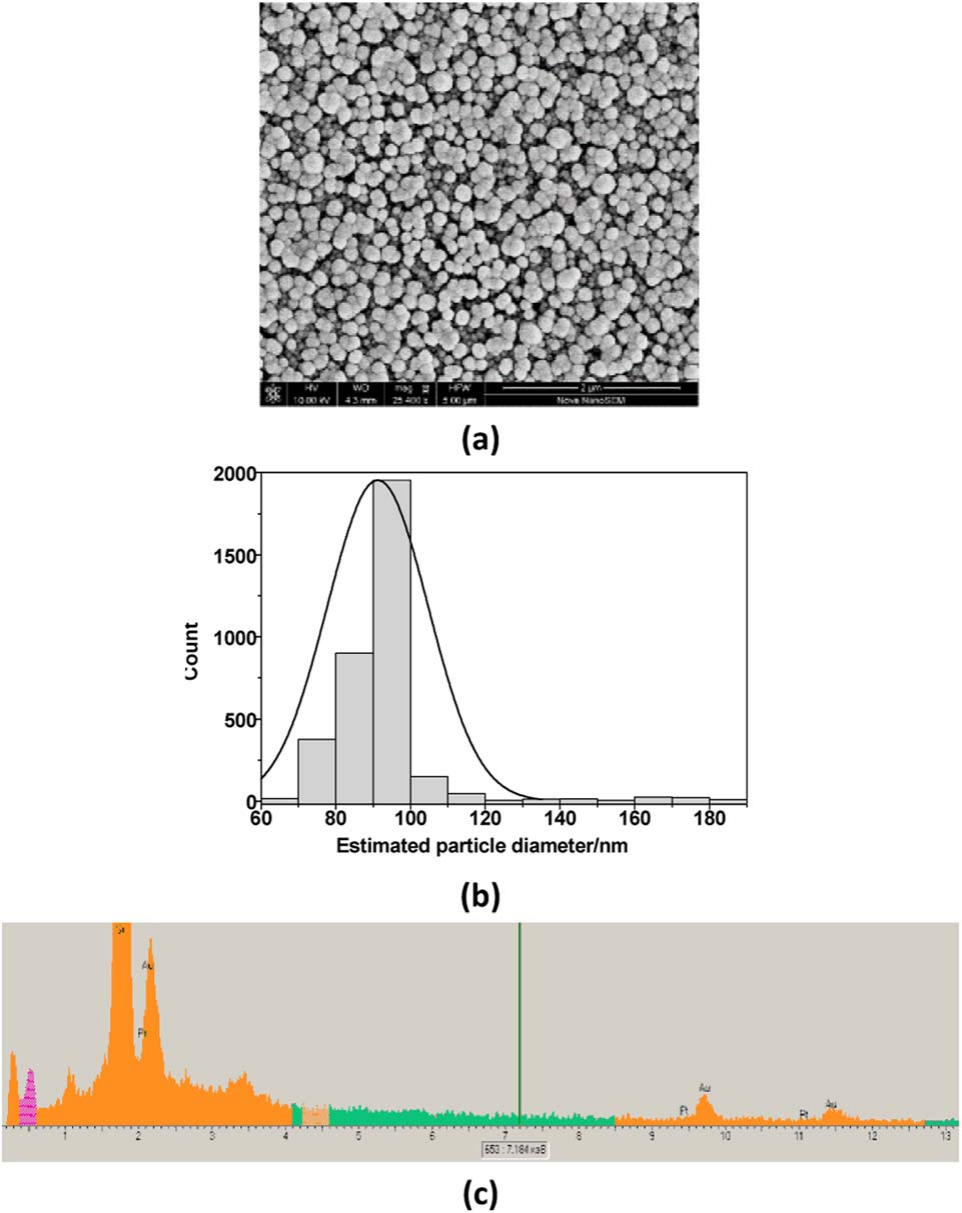
Morphological study of electrodeposited nAuPt on the surface of GCE: (a) – SEM image; (b) – the particle size distribution of NPs electrodeposited on GCE, and (c) – X-ray spectral microanalysis.

The surface of GCE was additionally modified with Au and Pt nanoparticles. It is well known that the additional layer of Au or Pt nanoparticles is widely used in electrochemistry with the aim to increase the sensitivity of the sensors.^27–33^ In this work, a possible effect of additionally deposited layer of Pt and Au, covered by Nafion, on bioanalytical parameters of the fabricated sensors (LacNZs/nAuPt/GCE) was also investigated. SEM images and X-ray spectrograms were used to study the morphological and structural characteristics of the electrodeposited nAuPt *in situ* (Fig. 3). The generated nAuPt layer was confirmed by SEM to be nanosized with an average particle size about 90 nm (Fig. 3). The X-ray spectral microanalysis of the nAuPt show distinctive peaks for Au^0^ and Pt^0^ (Fig. 3b).

The main bioanalytical parameters of the constructed LacNZs/nAuPt-based sensors, namely nPtCu/nAuPt/GCE and nAuCu/nAuPt/GCE have been studied. Fig. S5† demonstrates a higher current signal at +200 mV and above for nAuCu/nAuPt/GCE.

**Fig. 4:**
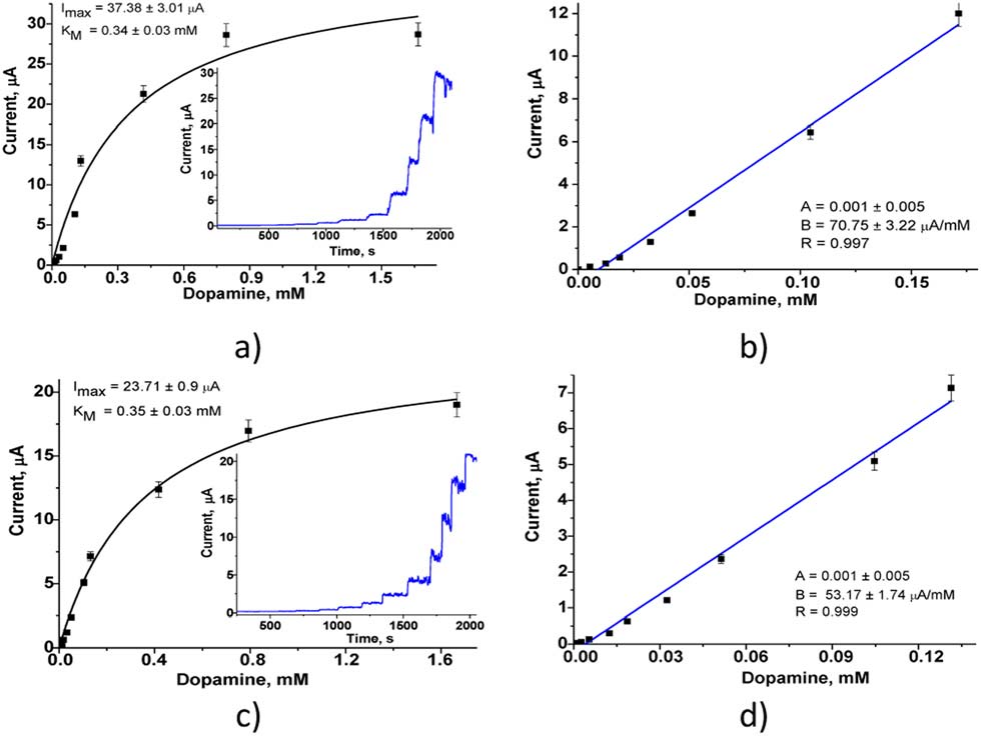
Amperometric responses during consecutive addition of DA in different concentrations. Calibration curves the sensors nAuCu/nAuPt/GCE (a and b), nPtCu/nAuPt/GCE (c and d) in PB (pH 7.0), *vs.* Ag/AgCl.

The amperometric characteristics of the constructed nPtCu/nAuPt/GCE and nAuCu/nAuPt/GCE sensors at the added concentrations of DA under continuous stirring are presented in Fig. 4. These nanosensors reveal a significant increase in sensitivity (3.0–3.6-fold; ·7540 A M^−1^ m^−2^ and 10 065 A M^−1^ m^−2^) compared with the electrodes without using nAuPt layer (nAuCu/GCE and nPtCu/GCE, respectively). The electrodes show a good linearity (1–130 μM and 1–170 μM), and their detection limits are 0.3 μM and 0.5 μM which are better than for amperometric sensors described in the literature (Table S1†). The prepared sensors exhibit a wide linear operating ranges as well as a low detection limit in comparison with the described nanozyme-based sensors (CuNCs–Gr/SPCE, MnO_2_/GQD, hemin-doped HKUST-1/rGO) and laccase-based bionanosensors (Lac–GA–NH_2_C_2_H_4_S, Lac–HNT–ImS_3_–14/CPE, Lac–Glu–AuNPs/CPE).^34–40^ The sensor described here, nAuCu/nAuPt/GCE has a 60-fold higher sensitivity compared to Hemin-doped HKUST-1/rGO and Lac–GA–NH_2_C_2_H_4_S–AuNS/GC sensors.^34,36^

The combination of nAuPt layer with laccase mimetics results in the oxidation of dopamine on the surface of the modified electrode at a lower applied potential, creating a higher current density and better conductivity, which leads to an increasing sensitivity of the sensor, while lowering the detection limit. Possibly, it can be explained by synergistic effect of nAuPt additional layer on electron transfer rate between the artificial nanozyme and the electrode surface. The electrode nAuCu/nAuPt/GCE with a higher sensitivity can be a promising nanosensor for detection of DA.

### Reproducibility, stability and interferences for DA determination using nAuCu/nAuPt/GCE

**Fig. 5:**
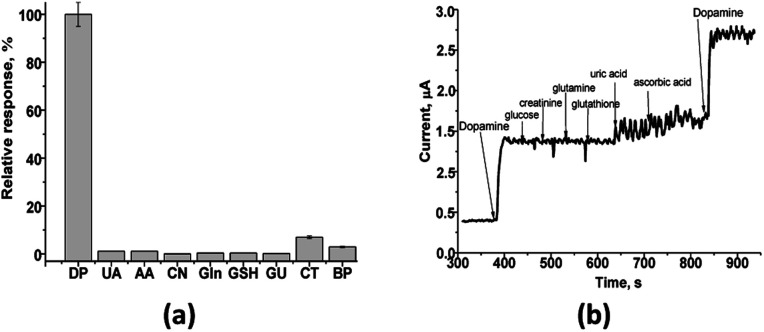
Characterization of nAuCu/nAuPt/GCE sensor: selectivity (a) and possible interference effect (b) for different compounds without and with the addition of 0.2 mM dopamine (DA). Glucose (GU), creatinine (CN), glutamine (Gln), glutathione (GSH), uric acid (UA), ascorbic acid (AA), catechol (CT) and bisphenol (BP). The analytical signals under the working potential +200 mV are presented in relative units (%) related to the maximal current signal on DA (100%).

The important parameters in determining a biosensor's suitability are selectivity, reproducibility, repeatability, and stability. Since many chemical components are present in real samples with dopamine, they can effect on its analysis. Therefore, the fabricated sensor's response was tested to different compounds, usually presented in biological liquids, namely, glucose (GU), creatinine (CN), glutamine (Gln), glutathione (GSH), uric acid (UA) and ascorbic acid (AA) (Fig. 5a). It was shown (Fig. 5) that the amperometric responses obtained under addition of uric acid, ascorbic acid, catechol (CT) or bisphenol (BP) are of a low value and thus do not interfere with the detection of DA in real samples.

The test on the selectivity of the developed nAuCu/nAuPt/GCE sensor was evaluated for added individually different compounds in 0.2 mM concentration. Only a low current response was observed for uric acid (UA) – 1%, ascorbic acid (AA) – 1% (Fig. 5 b).

To study the repeatability of the nAuCu/nAuPt/GCE sensor, we carry out 10 successive analyses of 0.25 mM DA in 0.05 M PB (pH 7.0) during one day. The relative standard deviation of the response of the constructed electrode was 4.1% which demonstrates a good repeatability for nAuCu/nAuPt/GCE sensor.

In our study, the seven separate nAuCu/nAuPt/GCE sensors have been fabricated to investigate their reproducibility of analytical date. The responses of the constructed electrodes were tested chronoamperometrically under injection of 0.2 mM DA. The relative standard deviation (RSD) was shown to be 3.1% which demonstrates a good reproducibility for the tested sensors.

To evaluate the storage stability of the nAuCu/nAuPt/GCE for 30 days, we measured the current responses for injected 0.2 mM DA daily under the same conditions. The results showed that after 14, 21, and 28 days, the responses of the electrode to DA were 97, 98, and 95% of the initial value, respectively. Therefore, the nAuCu/nAuPt/GCE sensor has a proper stability. Thus, the developed nanosensors may be advantageous for practical applications due to the easiness of their fabrication scalability, reproducibility and stability.

### Application of the fabricated amperometric LacNZs-based sensor for DA assay in pharmaceuticals

**Fig. 6:**
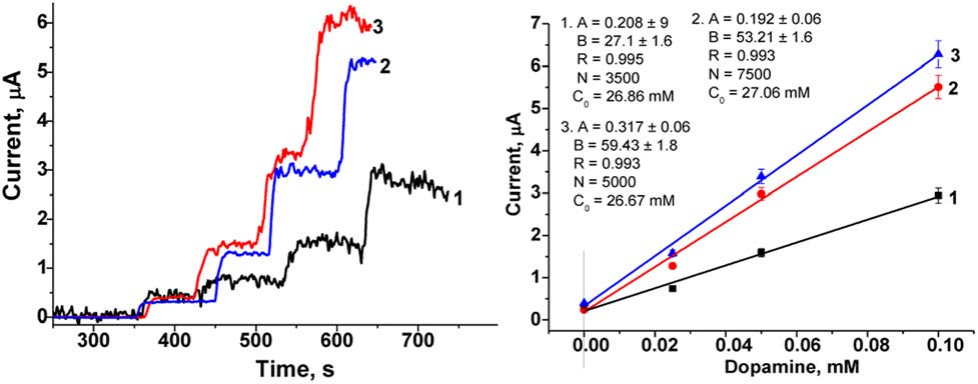
The estimation of DA concentration in the pharmaceutical dopamine hydrochloride using the nAuCu/nAuPt/GCE sensor and the standard addition method.

To verify the practical applicability of the developed nanosensor, the nAuCu/nAuPt/GCE was used to determine DA in real drug – dopamine hydrochloride ampoule for injection (Darnytsia, Ukraine). Each assay, being performed for tree dilutions of the sample, was repeated 3 times. The obtained results reveal a good DA recovery (94.1%). RSD (*n* = 3) values were 2.1–5.5% and a standard deviation of less than 1% confirmed the accuracy of LacNz-based sensor to detect dopamine (Fig. 6).

## Experimental

### Reagents

Copper(ii) sulfate, cobalt(ii) chloride hexahydrate, gold(iii) chloride solution, chloroplatinic acid, cerium(iii) hydrocarbonate, iron(iii) chloride, dopamine, ascorbic acid, Nafion (5% solution in 90% ethanol), 2,2′-azino-bis(3-ethylbenzthiazoline-6-sulfonic acid) (ABTS), dipotassium hydrogen phosphate, potassium dihydrogen phosphate, glutaraldehyde (25%), cetyltrimethylammonium bromide (CTAB), and all other reagents and solvents used in this work were purchased from Sigma-Aldrich.

### Synthesis and characterization of laccase-like NZs

The nCuMnCo, nPtCu, nAuCu were obtained by hydrothermal method. nCuMnCo was synthesized as follows: 5 mL 25 of mM MnSO_4_ were mixed with 0.1 mL of 1 mM CTAB. Then, an aqueous citric acid solution (100 mM, 1 mL) was added to the mixture and stirred for 30 min at room temperature, observing a color change from pink to brown. The product was precipitated *via* centrifugation at 10 000 rpm for 5 min and washed with distilled water. Later, 5 mL of 10 mM CuSO_4_, 5 mL of 5 mM CoCl_2_ and 0.5 mL of 10 mM ascorbic acid were added to the precipitate. The mixture was stirred for 5 min and transferred to an autoclave under high preassure (1 atm) at 120 °C for 1 h.

The nPtCu were obtained as follows: 1 mL of 5 mM H_2_PtCl_6_ were mixed with 0.1 mL of 10 mM CTAB, then 5 mL of 10 mM CuSO_4_ and 0.5 mL of 10 mM ascorbic acid were added while stirring for 30 min. After washing by centrifugation, the product was autoclaved at 100 °C for 1 h, and then cooled to room temperature.

To synthesize nAuCu, 5 mL of 1 mM HAuCl_4_ were mixed with 0.1 mL of 1 mM CTAB. Then 5 mL of 10 mM CuSO_4_ and 0.5 mL of 10 mM ascorbic acid were added while stirring and stirring was continued for 30 min. After washing, the product was autoclaved at 100 °C for 1 h.

The Co NPs were prepared by the chemical reduction method using NaBH_4_ as a reductive agent. 10 mL of 5 mM CoCl_2_ and 1.2 mL of 0.1 M NaBH_4_ were mixed and heated at 100 °C for 10 min with the following addition of 1 mL of 0.01 M CTAB to obtain a dark-brown suspension. nCo were deposited by centrifugation at 8000*g* for 30 min (Hettich Mikro-22R centrifuge), washed with 5 mM phosphate buffer, pH 7.0 (PB). To estimate the concentration of the formed NZ Co gravimetrically, the obtained precipitate was dried at 80 °C for 24 h.

For the obtaining of the Co (core)/Cu (shell) (nCoCu) NPs, the nCo were subsequently injected into the growth solution consisting of CuSO_4_ (5 mL, 10 mM) and CoCl_2_ (5 mL, 10 mM) with the following addition of NaBH_4_ (1 mL, 0.1 M) and heated at 100 °C for 10 min. The obtained NPs were precipitated from the solution by centrifugation at 8000*g* for 30 min and washed with 5 mM PB, pH 7.0 and water. The final nCoCuCe precipitate was dried (at 80 °C for 24 h).

Chemical synthesis of hexacyanoferrates (HCFs) was performed by mixing 2 mL of 50 mM K_4_Fe(CN)_6_, 2 mL of 50 mM CoCl_2_, 2 mL of 50 mM of CuSO_4_, and, finally adding 1 mL of 0.1 M citric acid.

All NZs were collected by centrifugation, washed with water, and stored as a suspension in water until use at +4 °C.

### Morphological characterization of nanocomposites

The morphology and size of nanoparticles were studied with scanning electron microscopy (SEM) FEI Nova NanoSEM 450 and SEM-microanalyser REMMA-102-02. The X-ray diffraction analysis of nanoparticles was investigated with STADI P X-ray powder diffractometer (“STOE & Cie GmbH”, Germany) with Cu Kα radiation (*λ* = 0.154 nm) for phase identification.

### Determination of pseudo-laccase activity

The laccase-like activity of synthesized nanoparticles was assayed by the use of ABTS as a chromogenic substrate.^28^ For this, NZ preparation (1 mg mL^−1^) was added to 1 mM ABTS in 50 mM acetic buffer, pH 4.5 the initial reaction rate was calculated for the formation of ABTS cation radicals, monitored at 420 nm. One unit (U) of laccase-like activity was defined as the amount of NZs required to oxidize 1 μmol of substrate per one min at 30 °C.

### Isolation and purification of natural laccase

Laccase was isolated from the fungus *Trametes zonate* 1525. The cells were cultivated at 28 °C in YPD medium supplemented with 1% sucrose and 0.25 mM CuSO_4_ for 5 days. To remove the mycelia, the fungal culture was passed through filter paper, and a cultural liquid was used for enzyme isolation. The cultural liquid was subjected to fractional precipitation by ammonium sulfate. Salt saturation was standardized by using different levels (0–40, 40–60, and 60–80%) of ammonium sulfate. The protein precipitate of the last fraction was collected by centrifugation (12.000 rpm, 40 min, 4 °C), then suspended in 5 mL 0.05 M sodium acetate buffer, pH 4.5, and dialyzed at 4 °C against the same buffer using the dialysis bag with an exclusion limit of 12 kDa. Obtained laccase preparation with the activity of ≥10 U mg^−1^ was used for the research.

### Modification of GCE with AuPt NPs

Before biosensor preparation, the glassy carbon working electrode (GCE) was polished with a polishing cloth using decreasing particle sizes of alumina paste. To deposit of AuPt NPs on the GCE surface, the electrodes were sonicated for about 5 min in different solvents (isopropanol, absolute ethanol, and deionized water). The cleaned electrodes were immersed in a solution containing 1 mL of 1.0 mM HAuCl_4_, 1.0 mL of 1.0 mM H_2_PtCl_6_ and 5.0 mL of 0.5 M sulfuric acid. Electrodeposition was performed by cyclic voltammetry with a potential range from +1.00 V to −0.3 V *vs.* Ag/AgCl/3 M KCl at scan rate of 25 mV s^−1^ during 10 cycles.

### Construction of Lac-NZs-based sensors

The constructed NZs-based amperometric sensors were characterized using amperometry in a three-electrode configuration with an Ag/AgCl/KCl (3 M) reference electrode, a Pt-wire counter electrode and a working GCE (an electrode area of 7.06 mm^2^).^27,28^

To construct nanostructured electrodes, LacNZs were immobilized on GCE, modified (or nonmodified) by AuPt NPs. For the immobilization of LacNZs, an aliquot of NZs (3 μL, 0.2 mg mL^−1^) was dropped on the surface of the GCE or AuPt NPs/GCE electrodes and dried. Then aliquot of 1% Nafion was dropped to form its film. Finally, the functionalized electrodes LacNZs/GCE or LacNZs/AuPt NPs/GCE were rinsed with 50 mM PB, pH 7.0.

### Measurements and calculations

All electrochemical measurements were conducted with an electrochemical workstation Metrohm Autolab PGSTAT30 with Ag/AgCI/3 M KCI electrode and platinum wire as reference and counter electrodes, respectively. All experiments were carried out in triplicate trials.

Analytical characteristics of the electrodes were statistically processed using OriginPro 10.5 software. Error bars represent the standard error derived from three independent measurements. Calculation of the apparent Michaelis–Menten constants (*K*^app^_M_) was performed automatically by this program, according to the Lineweaver–Burk equation.

### Determination of DA in pharmaceuticals

The pharmaceutical formulation “Dopamine solution for injection, ampoule” (“Darnytsia”, Kyiv, Ukraine) was used. The manufacturer has declared the following composition of this pharmaceutical product: 5 mg mL^−1^ dopamine hydrochloride in water for injections, hydrochloric acid and sodium metabisulfite (E223) in water for injections. Each assay, performed for two or three dilutions of the sample, was repeated 3 times.

## Conclusions

The current study reports the preparation of new nanozymes (nAuCu, nPtCu, nCuMnCo, nCoCuCe, nCuHCF, and nCoHCF) possessing laccase-like activity and their applicability for construction of amperometric nanosensors for dopamine assay at low working potentials. All constructed sensors based on the laccase mimetics showed a higher sensitivity (2-fold) compared to biosensor based on the natural laccase. The best laccase-mimicking nanozymes nPtCu and nAuCu were used for construction of sensors for dopamine detection. An additional modification of glassy carbon electrode with the AuPt NPs was also used to enhance sensors sensitivity. The constructed nanosensor with the architecture nAuCu/nAuPt/GCE demonstrated the highest sensitivity (10 650 ± 8.25 A M^−1^ m^−2^), a broad linear range (10–170 μM) and a lowest detection limit (20 nM DA). It showed a long-term storage stability and insensibility to the many interfering compounds. The created sensor was tested for analysis of dopamine in pharmaceutical dopamine hydrochloride, proving the reliability of the constructed device. This research may provide the way for the development of portable dopamine sensing modules and help to understand the correlation between dopamine level in biological liquids and the progression of neurodegenerative disorders.

## Author contributions

Demkiv Olha, Nataliya Stasyuk, Wojciech Nogala, Mykhailo Gonchar: investigation, validation, writing – original draft; Demkiv Olha, Nataliya Stasyuk: visualization. Halyna Klepach, Taras Danysh: methodology, visualization. Mykhailo Gonchar and Wojciech Nogala: conceptualization, supervision, writing – review & editing.

## Conflicts of interest

There are no conflicts to declare.

## Supplementary Material

RA-014-D3RA07587G-s001
