# Supplementary material for: Highly sensitive amperometric sensors based on laccase-mimetic nanozymes for the detection of dopamine
Source: RSC Adv. 2024 Feb 13;14(8):5472–8. doi: 10.1039/d3ra07587g (PMC10862099; doi:10.1039/d3ra07587g)
Supplement: RA-014-D3RA07587G-s001 [file RA-014-D3RA07587G-s001.pdf]

Electronic Supplementary Material (ESI) for RSC Advances.
This journal is © The Royal Society of Chemistry 2024

Electronic supplementary information

Highly sensitive amperometric sensors based on laccase-mimetic nanozymes for the detection of dopamine

Olha Demkiv[a*], Wojciech Nogala[b], Nataliya Stasyuk[a], Halyna Klepach[c], Taras Danysh[d], Mykhailo Gonchar[a,c]

[a]*Institute of Cell Biology, National Academy of Sciences of Ukraine, Lviv 79005, Ukraine.*
[b]*Institute of Physical Chemistry, Polish Academy of Sciences, 01-224 Warsaw, Poland.*
[c]*Drohobych Ivan Franko State Pedagogical University, Drohobych 82100, Ukraine.*
[d]*Institute of Blood Pathology and Transfusion Medicine, National Academy of Medical Sciences of Ukraine, Lviv 79044, Ukraine.*

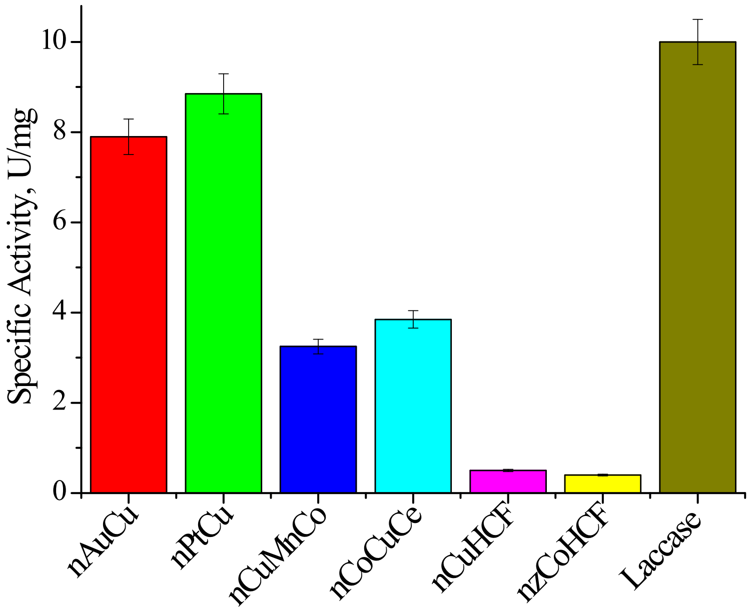

Fig. S1 Laccase-like activities of the obtained LacNZs

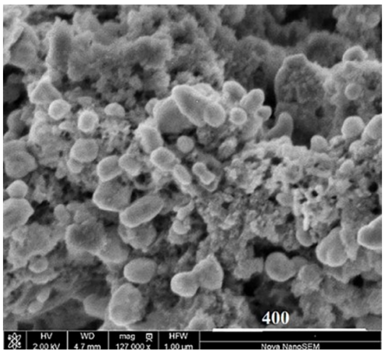

(a)

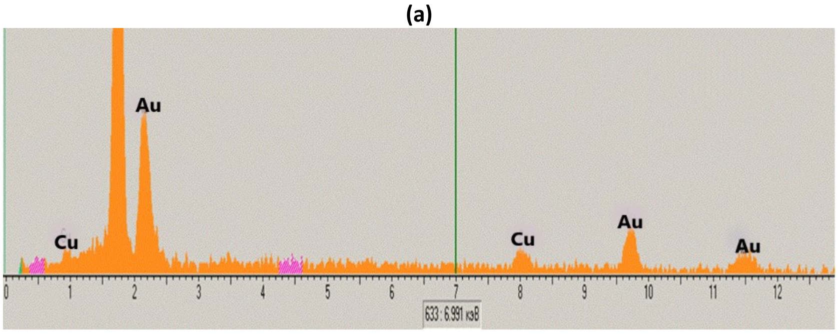

(g)

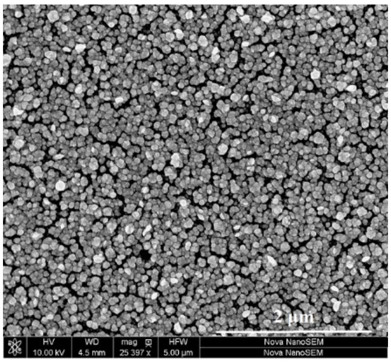
2 μm
HV 10.00 kV
WD 4.5 mm
mag 25 397 x
HFW 5.00 μm
Nova NanoSEM

(b)

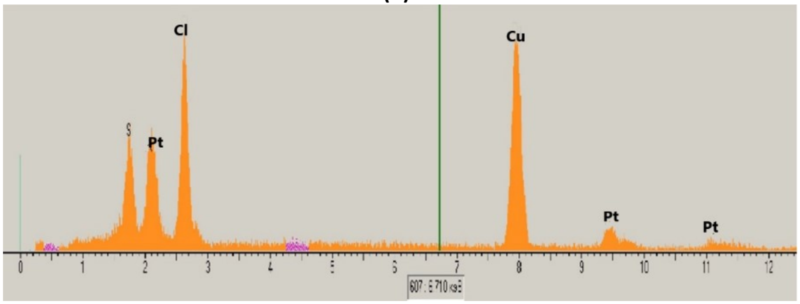
Cl
S
Pt
Cu
Pt
Pt
0 1 2 3 4 5 6 7 8 9 10 11 12

(h)

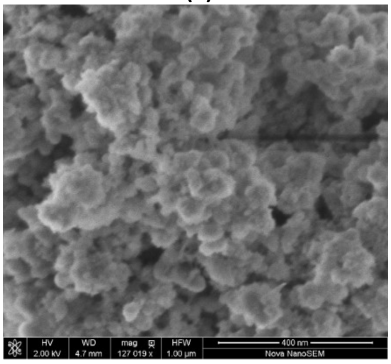
HV 2.00 kV
WD 4.7 mm
mag 127 019 x
HFW 1.00 μm
400 nm
Nova NanoSEM

(c)

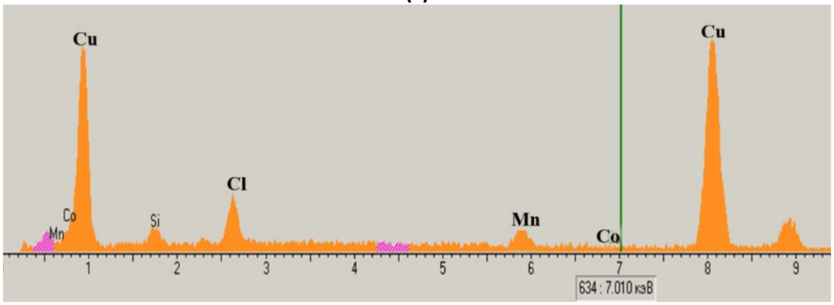
Cu
Co
Mn
Si
Cl
Mn
Co
Cu
1 2 3 4 5 6 7 8 9
634 : 7.010 кэВ

(l)

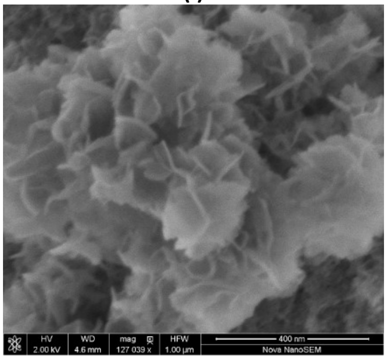
HV 2.00 kV
WD 4.6 mm
mag 127 039 x
HFW 1.00 μm
400 nm
Nova NanoSEM

(d)

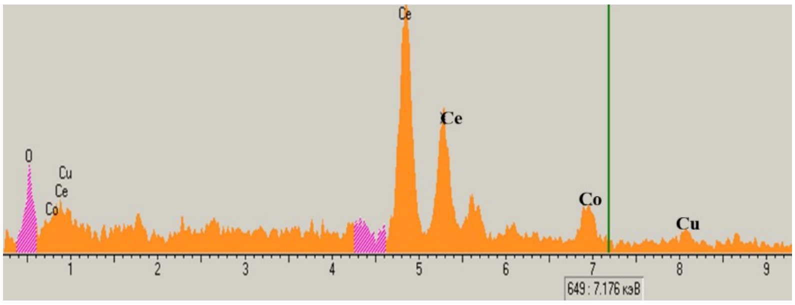

(J)

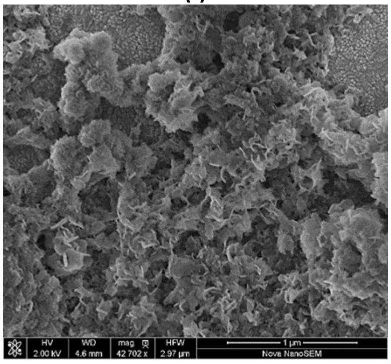

(e)

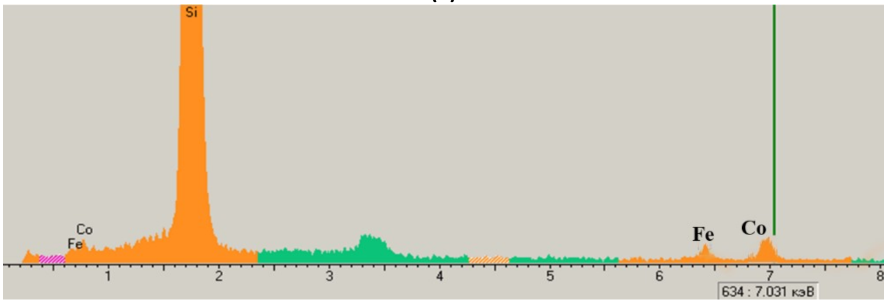

(k)

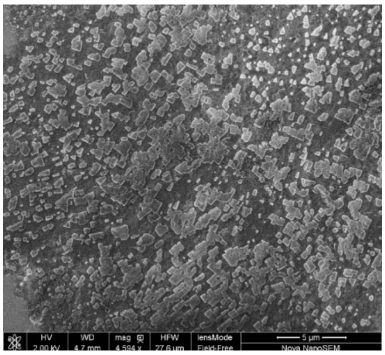

(f)

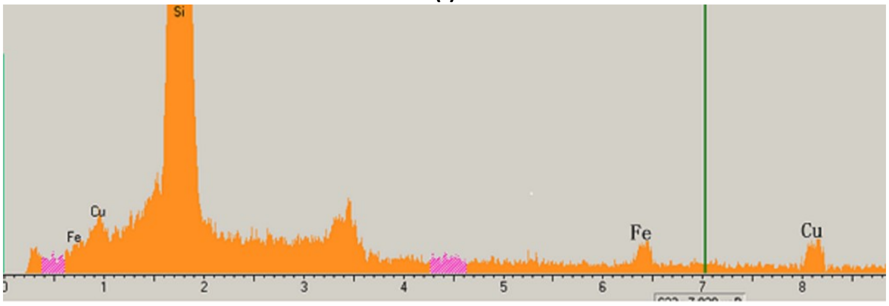

(L)

Fig. S2 SEM images (a, b, c, d, e, f) with X-ray microanalysis (g, h, i, j, k, l) of NZs, wich were obtained by the hydrothermal approach (nAuCu nPtCu,nCuMnCo,) chemical reduction method (nCoCuCe), and the method of drop-synthesis by mixing aqueous solution of $K_3Fe(CN)_6$ and $CoCl_2$ or $CuSO_4$.

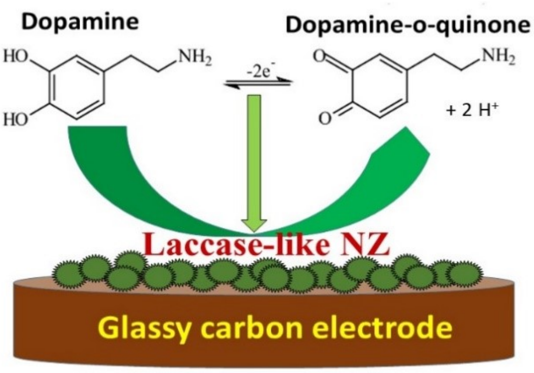

Fig. S3 Hypothetical scheme of the functionality of the laccase mimetics-based sensors.

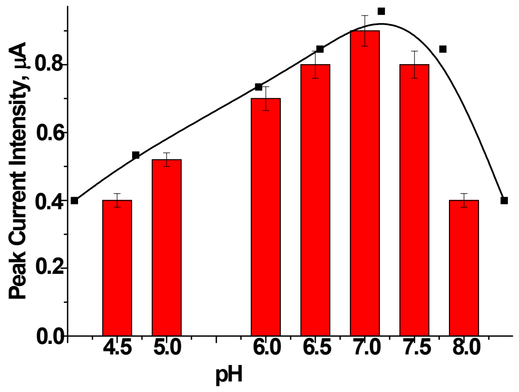

Fig. S4 Effect of pH on the DA-signal of nAuCu-based sensor

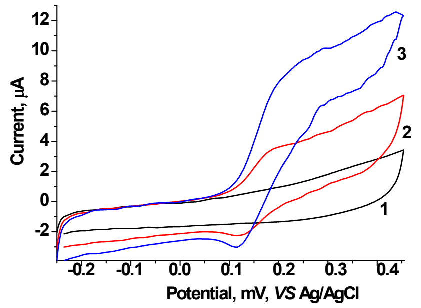

Fig. S5 The cyclic voltammogram for unmodified (1) and GCE-modified by nAuCu/nAuPt/GCE (2) in PB, pH 7.0 with added concentrations of DA: (1) — 0 mM; (2) — 0.1 mM; (3) — 0.2 mM at the scan rate of 25 mV·s^{-1}.

Table S1 Analytical characteristics of the fabricated nanozyme-based sensors for dopamine assay.

Sensor	Linear range, µM	LOD, µM	Sensitivity, $A \cdot M^{-1} \cdot m^{-2}$	References
nAuCu/nAuPt/GCE	2-175	0.0003	10650 ± 8.25	This work
nPtCu/nAuPt/GCE	3-130	0.0005	7540 ± 6.51	
nAuCu/GCE	8–80	0.008	2800 ± 1.89	
nPtCu/GCE	8–40	0.009	2550 ± 1.93	
nCuMnCo/GCE	3 - 30	-	1200 ± 1.65	
nCoCuCe/GCE	3-300	-	850 ± 0.71	
nCuHCF/GCE	3 - 300	-	1150 ± 1.29	
nCoHCF/GCE	3 - 300	-	1100 ± 1.79	
Laccase/GCE	5-150	-	378 ± 0.338	

CuNCs-Gr/SPCE	0.001–100	0.33 nM	196 mA·moL^{-1}	35
Co–N–C/rGA	3–2991	0.74	–	37
MnO_2 /GQD	0.5–100	0.05	–	38
Hemin-doped HKUST-1/rGO	0.03–10	0.033	1.224 μA μM^{-1}	36
Lac-GA-$NH_2C_2H_4S$-AuNS/GC	120	0.037	178 ± 5	34
Lac-HNT-ImS_3–14/CPE	0.99–67.8	0.252	–	40
Lac-Glu-AuNPs/CPE	0.8 – 62	6	–	39

CMC – carboxymethylcellulose; CPE – carbon paste electrode; CuNCs – copper nanocubes; GCE – glassy carbon electrode; Glu – glutaraldehyde; GOD – graphene quantum dot; HKUST-1 – also referred to as metal–organic frameworks (MOF)-199; a face-centered-cubic MOF containing nanochannels; HNT – halloysite nanotube; ImS3 – 3-(1-alkyl-3-imidazole)-propane-sulfonate; Lac – laccase; M-N-C – transition metal and nitrogen-doped carbon materials; NPs – nanoparticles
rGO – graphene oxide; SPCE – screen printed carbon *electrode.*
